# Pandemics, policy, and pluralism: A Feyerabend-inspired perspective on COVID-19

**DOI:** 10.1007/s11229-022-03923-4

**Published:** 2022-10-26

**Authors:** Karim Bschir, Simon Lohse

**Affiliations:** 1 University of St. Gallen, St. Gallen, Switzerland; 2 Institute for Science in Society, Radboud University, Nijmegen, The Netherlands; 3 Centre for Ethics and Law in the Life Sciences, Leibniz University Hannover, Hannover, Germany; 4 African Centre for Epistemology and Philosophy of Science, University of Johannesburg, Johannesburg, South Africa

**Keywords:** Epistemic pluralism, Scientific expertise, Policy advice, Evidence-based policy, Interdisciplinarity, Transdisciplinarity, Paul Feyerabend

## Abstract

We analyse insufficient epistemic pluralism and associated problems in science-based policy advice during the COVID-19 pandemic drawing on specific arguments in Paul Feyerabend’s philosophy. Our goal is twofold: to deepen our understanding of the epistemic shortcomings in science-based policy during the pandemic, and to assess the merits and problems of Feyerabend’s arguments for epistemic pluralism as well as their relevance for policy-making. We discuss opportunities and challenges of integrating a plurality of viewpoints from within and outside science into policy advice thus contributing to discussions about normative issues concerning evidence and expertise in policy-making.

## Introduction

In this paper, we use the case of the COVID-19 pandemic to contribute to discussions on evidence-based policy (Cartwright, 2009, 2013; Cartwright & Hardie, 2012; French, 2019; Khosrowi, 2019; Munro, 2015). More specifically, we analyse political responses to the pandemic by addressing the issue of *epistemic pluralism* in science-based policy-making through the lens of Paul Feyerabend’s philosophy of science. Our goal is to explore the question to what extent and in what ways the evidence base of policy can and should be pluralistic, i.e. rely on a multitude of epistemic perspectives or approaches. This characterisation of *epistemic pluralism* is deliberately broad and meant to encompass scientific disciplines, theories, and methodologies but also viewpoints informed by local expertise (the reasoning behind this decision will become clear below).^1^

While pluralism *within* science is a frequently discussed topic (see e.g. Kellert et al., 2006), and epistemic pluralism in evidence-based policy has already been addressed under the heading of “methodological pluralism” (Oliver & Pearce, 2017; Shlonsky & Mildon, 2014), the extent to which pluralism might have beneficial effects at the science-policy interface remains underexamined. In this regard, we believe, the philosophy of Paul Feyerabend provides a good starting point. Feyerabend was not only one of the most prominent philosophers of science of the twentieth century and an early proponent of a pluralist position, but his approach is also innovative in the sense that he draws implications from his pluralistic philosophy of science to the relationship between science and politics and the role of scientific expertise in society. Some of Feyerabend’s claims have, for good reasons, provoked strong criticism, and his arguments are not always taken seriously by philosophers of science. Nevertheless, we believe that Feyerabend’s texts contain important and valid arguments, which can, if properly reconstructed, help illuminate problems associated with epistemic hegemony in science in general, and in particular also in the context of science-based policy advice.

We will carve out the distinctive features of Feyerabend’s epistemic pluralism–in particular the idea of anomaly import between incompatible theoretical alternatives–and show how these features can be fruitfully applied within the context of science-based policy making. Our aim is twofold. On the one hand, we use Feyerabend’s pluralism to analyse the benefits and challenges of epistemic pluralism in policy contexts, and specifically in science-informed COVID-19 policies. At the same time, we intend to contribute to Feyerabend scholarship by highlighting the merits of Feyerabend’s arguments for the broader discussion revolving around science and policy, thus providing a philosophical-historical perspective on the relevance of Feyerabend’s arguments for current issues.

We will begin, in Sect. 2, by discussing some of the key aspects in the public reaction to the policy measures taken during the COVID-19 pandemic. We will distinguish two lines of criticism that have been brought forward against the ways in which science has informed and influenced policy-making, especially at the early stages of the pandemic. While criticisms within science mainly focused on the validity of specific knowledge claims, many public voices took issue with a lack of epistemic diversity in the response to the pandemic. We will focus on this deficit of epistemic pluralism. To this end, we lay out the arguments that underly Feyerabend’s pluralistic approach in Sect. 3. We show that Feyerabend’s pluralism grew out of his engagement with the main positions within the philosophy of science at his time, and how, at a later stage, he extended his pluralistic account beyond purely epistemological considerations to a controversial account of the role of science in free societies. In Sect. 4, Feyerabend’s arguments are applied to the scientifically informed policies implemented during the COVID-19 pandemic. This will serve to highlight the problems associated with a lack of pluralism in science-based policy-making and to flesh out the abstract arguments in concrete terms. Section 5 addresses objections to our analysis based on practical challenges for implementing more pluralism in policy-making. The concluding section provides a summary of the paper.

## Criticism of science-based COVID-19 policies

Rarely before has the importance of scientific policy advice been as evident as during the COVID-19 pandemic. Governments all over the world based their responses to the pandemic on science, often claiming that they were “following the science”. Epidemiological surveillance data and projections played a key part in this. In particular, theoretical projections by the COVID-19 response team at the Imperial College in London were a major factor in the implementation of lockdown policies in March 2020 (Adam, 2020). Many models predicted a massive overload of health care systems if no mitigation measures would be taken, which became an important driver for public health measures (especially non-pharmaceutical interventions, such as social distancing and lockdown strategies) around the world (for more details on other factors that influenced public health policy, see Jasanoff et al., 2021).

Scientists thus became key advisers for policy during the pandemic; they provided the evidence that was used to devise and revise public health measures and they played an important role in justifying these measures, either directly–in newspapers, on social media, and at official press conferences by state government–or indirectly, e.g. when politicians referred to scientific experts while explaining the reasons for a new set of restrictions to the public.

But there was also criticism of the way that science informed and guided policy-making during the pandemic, to which several scientists, philosophers of science and scholars of neighbouring fields contributed (e.g. Dupré, 2020; Fuller, 2020; Winsberg et al., 2020). Some criticised the quality and uncertainty of the available evidence and demanded more rigorous evidence standards for policy measures that were both extreme and unprecedented (Ioannidis, 2020).^2^ Value-laden assumptions, uncertainties and projections of epidemiological computer models were scrutinised or rejected by others (Chin et al., 2020; Saltelli et al., 2020). Finally, questions were raised about the transferability of projections of SARS-CoV-2 transmission dynamics and policy interventions between different countries (Sebhatu et al., 2020). These critical contributions revisit well-known themes in the philosophy of evidence-based policy, as they touch upon evidence quality and evidence hierarchies for policy, the roles of values and uncertainties at the science-policy interface, and the context-dependence of policy measures.

In public debates, however, another type of criticism played a central role. Many critical voices pointed at the strong focus on biomedical and epidemiological aspects as a guide for policy-making at the expense of alternative viewpoints and other types of knowledge, in particular regarding socio-economic aspects (e.g. Broadbent & Smart, 2020; Devlin & Boseley, 2020; Fore, 2020; Streeck, 2021). These criticisms allege that public health policies were predominantly based on the most recent biomedical findings on the modes of transmission of the virus (in particular at the beginning of the pandemic), theoretical models, and epidemiological indicators such as the 7-day incidence rate, the reproduction number R, and the aggregated number of infected people. In a similar vein, policy-makers were criticised for paying too much attention to experts from disciplines like epidemiology and virology. There was a widespread sense that little consideration was given to the societal aspects of the pandemic and the negative side-effects of drastic suppression strategies, reflecting the fact that public health strategies during the first waves of the pandemic, such as “flattening the curve” (The Economist 2020, February 29) and “the hammer and the dance” (Pueyo, 2020), were almost exclusively rooted in biomedical and epidemiological expertise. It seems that “follow the science”–even if taken with a grain of salt–was never an inclusive ideal, but only emphasised *some* disciplines, while marginalising others, especially the social sciences (see Lohse & Canali, 2021; see Kidd, 2021b on a Feyerabendian appraisal of “follow the science”).

To be sure, this does not mean that societal issues were completely ignored. In many countries, economic expertise was part of the policy advice process, and socio-psychological aspects were frequently considered by policy-makers.^3^ There is, however, mounting evidence to support the claim that there existed an *epistemic imbalance* in favour of a biomedical perspective and that policy-making was primarily based on biomedical and epidemiological evidence (see Jasanoff et al., 2021; Sell et al., 2021).^4^ COVID-19 task forces were dominated by medical experts and policy documents were predominantly informed by biomedical and epidemiological expertise. In addition, experts from medical fields played a much more prominent role in explaining and justifying public health measures to the public than social scientists or (oddly enough) public health scholars. Examples for this can be found in recordings of COVID-19 press conferences by the US government and many European governments, where biomedical experts and epidemiologists took centre stage on a regular basis.^5^

This lack of attention towards social scientific knowledge persisted throughout the pandemic and was not merely a relic of a rushed response in the beginning. Even though we were no longer in a position of an imminent and completely unknown health threat and the fall-out of lockdown strategies had become more apparent since late 2020, the biomedical perspective was still dominant, leading to a myopia in the *evidence* on which public health measures were based.

We argue that the various criticisms of the hegemony of biomedical and epidemiological evidence in policy-making share a common feature. They all point at *a lack of epistemic pluralism* in the management of the COVID-19 crisis. In the following, we analyse the problems associated with insufficient pluralism at the science-policy interface and discuss theoretical and practical arguments in favour of more pluralism. Drawing on Paul Feyerabend’s philosophy of science, we illustrate the concept of epistemic pluralism and its ramifications, and discuss normative implications for scientifically informed public health policy and policy-making more broadly.

## Feyerabend’s pluralism

To highlight the value of Feyerabend’s arguments for the assessment of the lack of epistemic pluralism in the management of the COVID-19 crisis, it is helpful to first look at the relevance of Feyerabend’s philosophy in twentieth-century philosophy of science more generally.

Determining the significance of Feyerabend’s work within academic philosophy of science is, however, a complicated task. Until recently only few professional philosophers of science have engaged with Feyerabend’s work.^6^ His influence on key themes such as values in science, the role of science in democracy and, most notably, pluralism, has not been fully examined. One obvious reason for this lack of attention within professional philosophy is the fact that the views Feyerabend brought forward and, in particular, the rhetoric styles he used to present them, were–to say the least–highly controversial. Feyerabend at times spoke rather unfavourably about philosophy of science as a field of intellectual inquiry and the people engaged in it. He frequently used *reductio* arguments to attack well-established positions of his peers and his writing often takes the form of an exuberant and obfuscating mixture of anecdotal examples, ironic remarks, wild exaggerations, and systematic philosophical argumentation.

Even though many contemporary contributions share important similarities with Feyerabend’s ideas, references to his works are made only fleetingly.^7^ Due to its (often hidden) influence on many important current contributions, it is worthwhile to reconstruct Feyerabend’s original position carefully and to situate it in contemporary discussions on pluralism. This will help to throw some facets of pluralism in relief that are sometimes neglected, in particular regarding the multitude of connections between the proliferation of epistemic approaches and empirical adequacy. While these connections are not entirely absent from the current landscape in philosophy of science, we believe they can be illuminated very fruitfully through the lens of Paul Feyerabend’s philosophy and can be used to further substantiate bridges to social epistemology, for instance concerning discussions on hermeneutical injustice and institutionalised epistemic blindness (Kidd 2019).

Feyerabend’s pluralism underwent significant changes over time. His first pluralistic account of science emerged in the context of his critical engagement with the philosophies of Karl Popper and Thomas Kuhn.^8^ In the later stages of this career, he applied the arguments he had used to support pluralism within science to the relationship between science and non-scientific traditions in society. It is therefore helpful to distinguish two stages of Feyerabendian pluralism: a theoretical stage and a political stage. While the former is focused on the epistemic benefits of pluralism for science, the latter provides a strongly normative account about the benefits of allowing and cultivating a multitude of positions and views within a free society.^9^

### Feyerabend’s theoretical pluralism

Feyerabend’s engagement with Thomas Kuhn’s theory of scientific revolutions (Kuhn, 1962) in the early 1960s, and in particular with Kuhn’s notion of “normal science” and the role of anomalies in revolutions, played a crucial role in Feyerabend’s first approaches to pluralism. A key problem for Feyerabend in this context was how anomalies become visible such that they can trigger paradigm changes and thus lead to scientific revolutions. In Kuhn’s view, revolutions emerge naturally, as it were, once normal science reaches its limits.^10^ This is where Feyerabend raises an objection. He thinks that, because scientists always may interpret certain experimental results in such a way that allows them to dismiss anomalies as irrelevant oddities, they are always rationally entitled to stick to their paradigm. He concludes that the only way to guarantee that normal scientific phases come to an end is to actively develop theoretical alternatives that are incompatible with the dominating paradigm.^11^ What he calls the “principle of proliferation” thus constitutes one of the core features of Feyerabend’s early pluralism.

He writes:

“Hence, if change of paradigms is our aim then we must be prepared to introduce and articulate alternatives, […] we must be prepared to accept a *principle of proliferation*. Proceeding in accordance with such a principle is *one* method of precipitating revolutions. It is a *rational* method” (Feyerabend, 1970, p. 205).

The idea that alternatives play an important and maybe even indispensable role in the test and potential falsification of established theories is also an essential element of Karl Popper’s critical rationalism.^12^ For Popper, no theory is infallible in all respects and scientific progress is achieved through the falsification of theories. Falsifications always require what Popper has called a “falsifying hypothesis”.^13^ Successful falsification necessitates an alternative theory that provides a possible explanation for why and how observed effects contradict the theory under scrutiny. Such theoretical alternatives can even be logically incompatible with the tested theory, i.e. they may contain statements that are false according to the theory under scrutiny.^14^ From this basic Popperian insight, Feyerabend derives the principle of proliferation, according to which scientific progress requires several competing alternatives within one domain of inquiry. For Feyerabend, pluralism is the normal, typical condition of a reasonably healthy unoppressed scientific community.

But Feyerabend goes beyond the Popperian point of emphasising the important role of alternatives in theory testing in that he also provides a mechanism of how theoretical alternatives can highlight anomalies and problems within established theories, which would remain undetected in absence of the alternatives. It is this element of Feyerabend’s pluralism that renders it unique in comparison with many current pluralistic accounts. It may be called the “anomaly importation thesis”.^15^ The central claim of the thesis holds that certain potentially falsifying counterinstances to a given theory become genuine counterinstances *only if* they are described from the vantage point of an alternative theory. Such alternatives can even be incommensurable to established theories.^16^ Theoretical alternatives–even incommensurable ones–can thus help to *unearth* counterinstances to any given theory. In Feyerabend’s words:

“[T]here also exist facts which cannot be unearthed except with the help of alternatives to the theory to be tested, and which become unavailable as soon as such alternatives are excluded. This suggests that the methodological unit to which we must refer when discussing questions of test and empirical content is constituted by a *whole set of partly overlapping, factually adequate, but mutually inconsistent theories*” (Feyerabend 1975b/2010, p. 27).

And:

“Hence the invention of alternatives to the view at the centre of discussion constitutes an essential part of the empirical method” (Feyerabend 1975b/2010, p. 29).

On this view, the cultivation of a plurality of alternative theoretical approaches becomes a necessary element for progress and a methodological imperative for science. To advance knowledge, scientists must develop, promote and cultivate theoretical and methodological alternatives to well-established paradigms.^17^ Pluralism thus acquires a crucial *epistemic* function.^18^

In *Against Method* (1975b/2010), Feyerabend combined this epistemological insight with a historical analysis. The history of science reveals that science always employed a variety of methods and theoretical approaches to achieve its aims. According to Feyerabend, the proliferation of theoretical alternatives as well as the violation of established methodological rules are important norms that were actually followed by successful scientists in the past. It is therefore not only descriptively inadequate but also methodologically wrong, to reduce science to a single method and to think of scientific progress as a continuing series of increasingly truth-like theories. Hence his infamous slogan “anything goes” (Feyerabend 1975b/2010), which must not be understood (as many have) as the dogma of some sort of unbounded epistemological anarchism, but rather as the ironic expression of a rational methodological “rule” that scientists have followed in the past.

### Feyerabend’s political pluralism

In later years, Feyerabend developed his pluralistic account further by integrating the epistemic role of a pluralistic methodology in science into a broader view of the importance of pluralism in society. From the late 1960s to the 1970s and onwards, Feyerabend engaged in a “critique of scientific reason” (see Feyerabend 1975a, 1976, 1978, 1980, 1987, 2011), in which he strongly criticised the dominance of the scientific worldview. In his (arguably most controversial) book *Science in a Free Society *(1978), he argued that in a pluralistic and open society, institutionalised science and its underlying materialist-rationalist worldview constitute only one of many legitimate traditions and thus deserve no privileged status in a democratic society.^19^ Feyerabend even went as far as to call the prevalence of the scientific worldview a threat to democracy (Feyerabend, 1978, Ch. II.2). For the sake of freedom, autonomy, and humanism, science should be separated from the state like the church is separated from the state (Feyerabend, 1978, Ch. II.10).^20^

The critique of scientific reason also includes a critique of the role of scientific expertise in society. Scientific experts, Feyerabend claims, are not entitled to a special epistemic or cultural authority. Ordinary citizens must supervise science and they have the right to criticise or even reject scientific claims, especially when such claims are used in public decision-making.^21^ The background of these radical and unorthodox claims is again the idea that in a free society, plurality of opinion, open debate and tolerance between incompatible traditions lead to better epistemic outcomes. Assigning any sort of special authority to scientific claims hinders, according to Feyerabend, the free exchange of views and ideas.^22^

Unsurprisingly, Feyerabend’s straightforward denial of any sort of special epistemic authority for science has led to harsh criticisms of his position and accusations of relativism (see e.g. Theocharis & Psimopoulos, 1987; Sokal & Bricmont, 1998). However, if one focuses on the actual arguments that are buried under a thick layer of provocations and exaggerations, one may recognise that the aim of Feyerabend’s attack on the epistemic authority of science is neither to deny that science significantly contributes to our understanding of reality, nor to put science on the same epistemic level as other traditions. Feyerabend merely, but all the more vehemently, argues against the hegemonic pretentions and bigotry that he ascribes to at least some proponents of the rationalist-scientistic worldview. His attacks are thus not so much aimed at the denial of the importance of science, but rather *against* the devaluation and abasement of non-scientific views in the name of science and rationalism. The core of his criticism consists in the claim that non-scientific approaches to reality and life can provide valuable insights.^23^

Feyerabend’s anti-hegemonic stance becomes even more clearly visible if one considers the strong influence that John Stuart Mill’s *On Liberty* (1859) had on Feyerabend’s account of a pluralist and open society presented in *Science in a Free Society*. Despite this clear and obvious influence, Feyerabend rarely quotes Mill and he hardly ever reproduces Mill’s arguments explicitly.^24^ Nevertheless, Feyerabend’s entire book may be seen as one long endorsement of Mill’s ideas. Lloyd (1997) provides a detailed analysis of the four reasons that Mill proposes in defence of his pluralistic account and that are constitutive of Feyerabend’s own account of the importance of alternatives and the free exchange of views and ideas. These reasons are:

1. The suppression of any views should be avoided because the truth might be among the suppressed.
2. The kernel of truth in minority opinions should be preserved for the good of everyone.
3. The rationality of any view is a function of its strongest competitors.
4. Any view can be better understood in the light of alternatives.

Analysing his account in the light of these Millian principles helps to understand that Feyerabend’s goal consists not so much in *devaluing* science (except, maybe, for reasons of provocation), but rather in *emphasising the value and importance of alternative views*, even those that may appear irrational or false from a strictly scientific perspective. It turns out that the same reasons that speak in favour of the proliferation of a plurality of theoretical and methodological approaches within science, also apply with regards to society more broadly and to the role that science and rationalism should play in relation to non-scientific approaches.

In essence, Feyerabend’s position may be seen as an anti-scientist critique of an excessive “expertocracy”. That is not to say that scientific experts should not play an important role in policy advice. But their expertise is not decisive and should not come at the expense of excluding relevant non-scientific perspectives (Feyerabend, 1982, pp. 86ff; cf. Sorgner, 2016). Thus understood, Feyerabend’s stance appears less crazy and more acceptable than it may seem at first sight.

We may now summarise Feyerabend’s pluralistic position as consisting of the following key elements:

- **Fallibilism**: No approach is infallible and every approach, while it might be highly useful to describe, explain, and predict certain aspects of empirical reality, is likely to fail in the description, explanation, and prediction of other aspects. It is therefore reasonable and epistemically virtuous to take a fallibilist stance even towards the most successful and broadly accepted scientific approaches.
- **Importance of alternatives and anomaly import**: Alternative perspectives allow for descriptions of relevant facts that would remain invisible and inaccessible in absence of the alternatives. By unearthing facts that can be relevant for the evaluation of a given approach, alternatives to that approach may help–in some cases they may even turn out to be necessary–to understand and uncover implicit assumptions and shortcomings of the latter.
- **Proliferation**: The proliferation and simultaneous application of a variety of approaches avoids a myopic picture that could arise if a single approach is applied to the solution of an epistemic problem.^25^
- **Science-transcending pluralism**: Extra-scientific forms of knowledge constitute important correctives to a strictly scientific perspective. They may provide important insights into aspects of reality that remain invisible from the perspective of the scientific worldview. Because these aspects may be highly relevant to the lives of people affected by science-based policies, those extra-scientific perspectives should not be excluded.

## Pluralism in public health policy

We now turn to the discussion of scientifically informed policy-making during the COVID-19 pandemic. We will analyse several examples in light of Feyerabend’s approach which will enable us to work out their significance and flesh out Feyerabend’s abstract arguments in concrete terms. The examples also serve to illustrate the fruitfulness of a Feyerabendian approach to scientifically informed policy-making. Before we do so, a few words are in order about why we think that Feyerabend's arguments are applicable here.

While Feyerabend originally developed his arguments for pluralism in the context of basic research, where scientists apply and test models and theories mainly for epistemic purposes, it is nevertheless important to note that, in certain situations, epistemological problems can be directly linked to policy-relevant practical problems. The COVID-19 pandemic constituted such a situation. Answers to scientific questions about the behaviour of the newly emerged virus and the dynamics of the pandemic had to be found while, at the same time, policies to fight the pandemic had to be implemented. New scientific insights potentially had a direct impact on those policies. In such a situation, epistemic problems and policy-problems merge. This is particularly apparent in the context of science-based policy advice, which “impose[s] political agendas by impelling practitioners to make decisions based on the *available* research” (Oliver & Pearce, 2017, p. 4, our emphasis). When scientific results have a direct effect on policy, unearthing epistemic shortcomings in the (use of) science that informs policy is crucially important, and philosophical arguments such as Feyerabend’s can be helpful in this regard.

We will organise the following discussion into subsections on (a) intradisciplinary, (b) interdisciplinary and (c) science-transcending epistemic pluralism.^26^ While *intradisciplinary epistemic pluralism* refers to the idea of relying on more than one school, research tradition, methodological approach etc. *within* a discipline, *interdisciplinary epistemic pluralism* refers to the idea of relying on more than one scientific discipline or area when addressing a problem. For example, criticising economists for not considering Marxist and other heterodox perspectives in the assessment of economic effects of global warming means criticising them for an insufficient degree of *intra*disciplinary pluralism, in this case internal to the discipline of economics. Criticising a social scientific analysis of the educational sector for not including economic evidence points to an alleged lack of *inter*disciplinary epistemic pluralism. *Science-transcending epistemic pluralism* concerns the issue of bringing scientific *and* non-scientific perspectives to the table of science-based policy. When labour policy is criticised for blindly following academic economists and not taking into account the practical insights of employers and employees, this may be understood as criticising policy-makers for a lack of science-transcending epistemic pluralism.

### Intradisciplinary epistemic pluralism

Feyerabend’s account clearly speaks in favour of intradisciplinary epistemic pluralism. According to Feyerabend, scientific disciplines should organise themselves in such a way that they allow for a multitude of methodologies and theoretical approaches when engaging with problems and questions that are relevant for that discipline. Feyerabend provides a unique argument in favour of intradisciplinary pluralism based on the principle of proliferation and his idea of anomaly importation. The crucial point here is that no single approach to a given problem is free from epistemic shortcomings and blind spots and that alternative approaches are sometimes necessary to unearth these shortcomings.

The strong focus on epidemiological modelling and *in silico* models during the COVID-19 pandemic constitutes a case in point for a myopia in the science-based advice that informed COVID-19 policies. As computer models always come with a certain degree of uncertainty, and errors in the estimates of initial conditions can lead to false predictions, policy-making might have been–and in some cases was indeed–influenced by inaccurate projections of likely COVID-19 scenarios (Saltelli et al., 2020; see also Lohse & Bschir, 2020).^27^

In fact, policy-directed modelling during the COVID-19 crisis did exemplify a certain degree of intradisciplinary pluralism. Modellers not only used one type of model but combined different types of models, such as agent-based and equation-based compartmental models that aimed to project likely pandemic dynamics conditional on different policy measures. If different types of models make roughly the same projections, this corroborates the reliability of the models (all things being equal). Yet, the usefulness of this approach is limited if the models in question do not really represent independent perspectives on their target systems (due to structural similarities), are calibrated with the same set of poor data, rely on the same set of (often highly uncertain) assumptions, or misrepresent the causal patterns underlying pandemic dynamics. All of these potential limitations of COVID-19 models have been discussed, throwing a sceptical light on the central role that computer models played for policy-making (Edeling et al., 2020; Friedman et al., 2020; Fuller, 2020).^28^ Through a Feyerabendian lens, one solution to deal with some of these issues would be to enhance model proliferation by introducing alternative approaches (cf. Veit, 2020).^29^ For instance, epidemiologists could not only use traditional SIR-models^30^ and models that are based on the same underlying rationale, but also consider models highlighting other aspects of pandemic dynamics such as effects of social networks for virus transmission (Manzo, 2020) and effects of behavioural feedback loops due to self-fulfilling or (-defeating) predictions (Tyson et al., 2020).^31^ Model proliferation can help to unearth prevalent but problematic assumptions of SIR-models–say *social network hubs do not have a strong effect on transmission dynamics in a pandemic*.

Insights from other approaches within epidemiology can also highlight limitations of epidemiological models in Feyerabend’s sense by revealing the relevance of socio-demographic facts that would remain invisible and inaccessible in absence of alternatives. Consider, for example, detailed socio-economic data on COVID-19-infected people who end up in an ICU. The collection and use of such data by social epidemiologists (which has only been done in some countries) can help to better understand which demographic groups get infected with a higher likelihood or have a higher likelihood for a severe development of the disease. Furthermore, epidemiological research can be deployed systematically (and used by local authorities) to assess in which settings people are likely to get infected, thereby, again, helping to reveal potential limitations of theoretical models and to discover ways to calibrate these for future pandemics (whose occurrence is, unfortunately, more likely than ever). All these examples point to ways in which intradisciplinary pluralism can be vital for improving the evidence-base for policy and facilitate better targeted and sensible measures to cope with pandemics.

### Interdisciplinary epistemic pluralism

Feyerabend not only provides arguments in favour of *intra*disciplinary but also for *inter*disciplinary pluralism. Considering a variety of perspectives from different scientific fields can be crucially important in the framing of specific epistemic problems. Describing a given problem from different disciplinary perspectives can help to uncover aspects of the problem that would remain invisible were it approached from one and only one perspective. Again, the characteristically Feyerabendian insight behind this rests on the idea that uncovering epistemic shortcomings of any given approach *necessitates* alternative approaches. This also holds for problem framing. Problem analyses can vary depending on the disciplinary perspective from which problems are described, and the limitations of every single perspective become visible only if other problem descriptions are considered.

In the case of the COVID-19 pandemic, this amounts to the inclusion of non-medical perspectives, which are not only crucial for rational and effective policy interventions but can also help to uncover the restrictions and limitations of an epidemiology-centred view of the problem.

Here are a few examples to illustrate this point.^32^ There is a large body of social scientific work on issues that are highly relevant in the context of the COVID-19 pandemic (and indeed in the context of any pandemic). This includes work on job-related gender inequalities, insufficient participation of elderly people in society, structural racism, and inequality of educational opportunities for children from immigrant families. As we now know, there were policy-measures which led to a disproportionate deterioration of the situation for socially and socio-economically disadvantaged groups. Due to pre-existing gender stereotypes and job arrangements, women were much more burdened with childcare responsibilities during the pandemic (Power, 2020), elderly people living in nursing homes suffered from massively exacerbated isolation due to lockdowns (Abbasi, 2020), “environmental racism” (racial differences in exposure to pollutants) increased the likelihood of dying from COVID-19 for minority groups (Washington, 2020), and long distance learning solidified pre-existing social inequalities in schools (Sari et al., 2021). Effects like these were neither an accident nor a surprise for social scientists. The social sciences could undoubtedly have helped to better understand how the pandemic affects society on a socio-economic level (in the sense of a “syndemic”, Horton, 2020). They would also have been of use in predicting (at least qualitatively) unintended but likely side-effects of pandemic suppression strategies on different social groups and to develop targeted measures to cope with these side-effects–or to develop alternative suppression strategies. In short, and in a Feyerabendian vein, social scientific approaches could have made essential contributions to scientifically informed policy-making.^33^

In addition to this broadening of epistemic focus, inputs from the social sciences are also helpful for answering the question why some of the later scenario projections of the pandemic, for example in Canada and Switzerland, were off the mark. Social scientists have tools to analyse the effects of attitude and behavioural changes during the pandemic and investigate to what extent changes are a consequence of ineffective communication by governments, feedback effects of the models (see above), of general pandemic fatigue, and/or something else. By deploying these tools, the social sciences can indeed help to unearth facts that may be highly relevant for understanding limitations of epidemiological models that do not consider these social factors.

Interdisciplinary epistemic pluralism involving the social sciences is particularly important in the absence of truly pluralistic political representation (e.g. in European parliaments, in which upper strata of society and white-collar backgrounds are structurally over-represented, see Best, 2007; Elsässer et al., 2017). Adequate representation of all parts of society would mean that politicians should not only rely on personal (and selective) experience, conversations with particularly committed voters and lobbyists. Rather, what is needed is social scientific expertise as well as actual data regarding social aspects that is weighed on par with biomedical and epidemiological aspects. Otherwise, we have a situation that facilitates imbalanced policy-making: If we shine the brightest spotlight on medical aspects of a pandemic, but societal consequences can only be guessed at, what will politicians in a media democracy base their actions on? Different bodies of knowledge give rise to different policy choices.

The case for interdisciplinary epistemic pluralism is not limited to epidemiology and the social sciences. Consider the insights–initially viewed with scepticism–that aerosol scientists contributed to our understanding of the transmission of SARS-CoV-2. Their models provided strong evidence for the airborne transmission of the new coronavirus (see Morawska & Milton, 2020; Prather et al., 2020), a fact that remained unrecognised by policy-makers in 2020, who primarily relied on expertise from biomedicine.^34^ By deploying knowledge from aero- and hydrodynamics, and methods from engineering, such as laser-light scattering for droplet detection (Lewis, 2020), aerosol scientists were able to corroborate the relevance of airborne virus transmission. This approach has uncovered unwarranted assumptions of the received view, in particular that it is mainly larger droplets, not aerosols, that are responsible for the transmission of respiratory viruses over short distances (Greenhalgh et al., 2021; Tang et al., 2021). In this sense, and in accordance with the Mill-Feyerabend view, the limitations of the received view could indeed only be fully understood in light of an alternative.

Interdisciplinary epistemic pluralism not only improves the evidence base for policy decisions by revealing relevant facts and highlighting the limitations of prevalent approaches. It can also broaden horizons in terms of which ethical and political aspects need to be considered as relevant during an ongoing public-health crisis (see Gostin et al., 2020)–especially when it comes to understanding who will be affected by measures and which trade-offs must therefore be considered for different groups in society (cf. the discussion of “treatment effect heterogeneity” of evidence-based policies in Khosrowi, 2019). Without actual evidence, it is hardly possible for politicians to weigh different harms to be expected on different levels and for different groups, at least not in a well-informed way. In this specific sense, epistemic shortcomings are entangled with political injustices.

### Science-transcending epistemic pluralism

Feyerabend’s account not only provides arguments in favour of inner-scientific but also for science-transcending pluralism, as he does not restrict the domain of permissible epistemic alternatives to the sciences only. Quite the contrary, Feyerabend is maximally permissive when it comes to determine what might count as a viable alternative to currently preferred scientific approaches. He writes: “There is no idea, however ancient and absurd, that is not capable of improving our knowledge. The whole history of thought is absorbed into science and is used for improving every single theory” (Feyerabend, 1975b/2010, p. 33). Thus, the set of possible alternatives which fall under the principle of proliferation, and which could lead to the detection of shortcomings in established approaches, encompasses, in the limit, the entirety of human knowledge and imagination. It is worth noting that this is a distinctive feature of Feyerabend’s pluralism within the philosophy of science.

In the case of the COVID-19 pandemic, this means that a genuinely pluralistic policy approach should have included non-scientific perspectives and experts who provide local knowledge of relevant social spheres (e.g. nurses, caregivers in retirement homes, or teachers). This claim is particularly salient in case of unintended consequences of policy measures that have been implemented to suppress the pandemic. After the first and second waves of the pandemic, it became increasingly clear that many governments failed to take into account the multitude of negative impacts of lockdown measures on the lives of different social groups (Caduff, 2020; Joffe, 2021). This is in part a consequence of the medicine-centric understanding of the pandemic and the limited involvement of the social sciences in public health policy. However, it also seems to be an effect of a shortage of channels that were used to effectively integrate local knowledge (e.g. on domestic violence in specific communities or viable testing strategies in primary schools) into policy-making. Through appropriate channels for “civic participation” relevant information on social issues could have been made available more directly and in a timelier manner.

However, it is not only local knowledge in a strict sense that can be important in identifying latent aspects and hidden layers of the reality of public health crises. Consider the case of Long Covid. This term describes the phenomenon that a significant number of COVID-19 patients do not seem to fully recover and suffer from a persistent state of ill health for weeks and even months after the initial infection (Callard & Perego, 2021). While it is unclear how many people suffer from Long Covid,^35^ who is affected and why, or what the likelihood of full recovery is, it is clear how we became aware of this condition. Its relevance was primarily pointed out by those affected, in particular on the basis of detailed case descriptions of lingering symptoms after a COVID-19 infection on social media (see, e.g., #longcovid posts from mid 2020 on Twitter) and through patient stakeholder groups who advocated for the acknowledgement of Long Covid as a condition that deserves serious attention (Perego et al., 2020). Patient involvement was central here. It provided early case descriptions and established the use of “Long Covid” as a fixed term to recognise the suffering of people with an multifaceted and poorly understood but *real* medical condition (for the importance of this, see Carel & Kidd, 2017; Löwy, 2021). For these reasons, Long Covid can indeed be considered a “patient made illness” (Callard & Perego, 2021). The involvement of patient groups was also a driving force for making the significance of Long Covid *visible* for public health authorities, including the WHO, thereby providing essential information for short-term suppression strategies and long-term public health planning. In this sense, science-external perspectives were not only providing “data points” for the biomedical sciences. Rather, they (in a Feyerabendian spirit) challenged prevalent assumptions regarding disease severity and likely public health effects that were primarily informed by experts from biomedicine. In doing so, they brought a hidden dimension of COVID-19 to light and called into question the overreliance on established evidence hierarchies in science.

Another example for the importance of extra-scientific sources of information are nursing homes. A survey conducted by the administration of an organisation that runs several nursing homes all over Switzerland hosting over 5000 elderly persons found that a significant number of inhabitants expressed the wish not to be transferred to a hospital should they contract COVID-19. In rural regions the fraction of elderly people who preferred to be treated and, if it need be, die in the nursing home instead of a hospital reached up to 90%.^36^ A report by the Swiss Federal Office of Public Health also found that roughly 50% of COVID-19 deaths in Switzerland between October 2020 and February 2021 occurred in elderly or nursing homes.^37^ The fact that a large number of elderly people prefer not to be hospitalised is certainly an important piece of information for policy makers if they have to estimate hospital and ICU capacities. Taking into account the fact that a certain number of patients above a certain age is likely to refuse hospitalisation when infected, can lead to more realistic estimates of when hospital capacities reach their limits. However, this type of information only becomes available fast enough if one has direct access to the local knowledge of nursing home administrations and if that knowledge is included in the evidence base for policy-making.

To be sure, evidence of this sort cannot be judged by the same standards as scientific evidence as it is usually not generated in a process that satisfies the rigor of a properly conducted empirical study.^38^ But even if it does not undergo the same vetting process as scientific evidence, evidence from extra-scientific sources can play an important role in highlighting potential blind spots in the models used by scientists. In contrast, the more rigidly evidence standards are interpreted in the sense of a traditional understanding of evidence-based policy, the more likely it is that relevant aspects–those for which there is yet no sufficiently robust data–will be ignored. Again, there is an entanglement of epistemic and political aspects in public health policy.

This is precisely the argument that Feyerabend uses in favour of his permissive stance towards non-scientific forms of knowledge. Extra-scientific perspectives should be included, not because they are closer to the truth than science or because their evidence satisfies the same standards as the evidence produces by science, but because they may point at limitations in the strictly scientific approaches that would otherwise remain invisible. For Feyerabend, society benefits if its decisions and policies are not based on insights from science alone. The extra-scientific sources of information that did or could have improved policies during the COVID-19 pandemic are an excellent example for that.

## Critical discussion

The scope of our discussion so far was clearly on public health policy in times of crisis. The question thus arises to what extent the arguments presented in this article also hold for public health policy more broadly or even for science-based policy in general. While many problems associated with a lack of pluralism in the response to the COVID-19 outbreak are generally recognised and well-understood, the crucial questions that should be asked thus are: How should we design *future* science-based policy-making in the context of public health threats*?* And what are the general requirements for more pluralism in policy advice?

At this point, it is important to recognise that Feyerabend’s arguments for pluralism are mainly negative, as they are often framed as counterarguments *against* philosophical accounts that promote methodological hegemony or other forms of epistemic monism. As valuable as his arguments may be for highlighting the shortcomings and inconsistencies in these accounts, they are, unfortunately, less helpful when it comes to developing a constructive and positive account of how pluralism can be implemented.^39^ In practice, promoting epistemic pluralism raises numerous challenges. In light of this, we conclude by briefly discussing three objections to epistemic pluralism in policy contexts.

The *pragmatic* objection claims that increasing epistemic pluralism is likely to inhibit evidence-based policy in practice. Pluralistic deliberation takes time, a resource that is often scarce, especially during a crisis. But even when time is not an issue, a greater diversity of perspectives, approaches and epistemic standards decreases the likelihood that consensus positions for policy advice are found, as every majority statement would be accompanied by a non-negligible number of minority votes. However, decision-makers may hardly benefit from being exposed to an unmanageable multitude of policy options. In addition, the communication of (too much) dissent to policy-makers may negatively affect the credibility of policy advice.

Although this issue poses a serious obstacle to pluralist evidence-based public health policy, this does not mean that epistemic pluralism is neither desirable nor feasible. Rather, it means that these obstacles need to be deliberately addressed in a practice-oriented fashion to create more pluralistic platforms at the interface of science and policy as well as feasible ways of condensing and communicating dissent and uncertainty. This can be done, for example, by experimenting with new ways of pluralistic deliberation, consensus finding and science communication. The rich body of literature on participatory governance, best practice models for deliberative decision-making and science communication in policy contexts points into the right direction (e.g. Bielak et al., 2008; Jamieson et al., 2017; Levi-Faur, 2012, part VII; for a recent contribution in the context of COVID-19 see Norheim et al., 2021). However, there currently exists no unified model for a truly pluralistic evidence-based policy-making. The greatest challenge at this point in time consists in merging useful ideas from various fields of inquiry into a workable and efficient framework that increases the degree of epistemic pluralism in evidence-based policy.

A second objection concerns inter- and transdisciplinary knowledge integration. Let us assume we were able to select a team of medical experts, social scientists as well as experts on relevant local knowledge in a public health policy setting. Would they be able to understand each other and communicate effectively? What is more, would they be able to agree on what counts as relevant pieces of evidence and how different types of evidence should be integrated and weighted in policy advice? These questions point to deep issues of inter- and transdisciplinary understanding and evidence amalgamation in light of differences in evidence hierarchies across scientific disciplines and beyond. They pose another serious challenge for epistemic pluralism in evidence-based policy.

One way to address the underlying issues is by actively promoting scientific and transdisciplinary literacy and science communication skills, for example by (re-)introducing elements of a *studium generale* in graduate programmes. This would not only be helpful for understanding evidential claims from different fields but could also facilitate reflexivity regarding the limitations of different epistemic domains (including one’s own). An additional way to cope with challenges to inter- and transdisciplinary knowledge integration is to deploy moderators, i.e. people who are trained in different knowledge traditions and able to facilitate pluralistic interaction. They could chair deliberative policy-advice meetings by translating, posing the right questions, and balancing differences. Inter- and transdisciplinary scholars, philosophers of science and scholars of neighbouring meta-scientific fields might be well equipped to undergo such training programmes.

The last objection is a relative of the *demarcation problem* in philosophy of science. It can best be expressed by a simple question: Who should *not* sit on the table of science-based policy and why? We cannot have an unlimited number of voices in policy advice, so we would need some kind of cut-off and, most certainly, communities and associations that select representatives. But there is a deeper epistemological issue. Wouldn’t we need some kind of threshold for epistemic competence to decide who should and shouldn’t sit on the table of policy advice? It seems that, if we take Feyerabend seriously, this is not an option, as he is extremely liberal in terms of permissible epistemic traditions and vehemently criticises “expertism”. Does that mean that we would need to include virologists, sociologists, and experts for nursing homes as well as faith healers and conspiracy theorists? Surely this would also mean the end of evidence-based policy.

Our reply to this objection is twofold. On the one hand, it draws on our reconstruction of Feyerabend as defending a Millian position, according to which we should be maximally permissive, because even erroneous opinions may help to improve our epistemic picture. After all, having fringe positions represented does not mean that policy-makers will need to act on fringe claims. It just means that these voices should be considered in good faith before making policy recommendations, as they might contain a kernel of truth. At the same time, there must be limits—even for an epistemological anarchist. In particular, we would not be required to engage with what Feyerabend calls “cranks” (see Shaw, 2021b for an illuminating analysis of this notion in relation to Feyerabend's thinking). Cranks are actors who are only interested in pushing their own point of view and agenda. They do not want to learn from others, and they are not interested in questioning or revising their position in light of other views, evidence or arguments. Examples for cranks are conspiracy theorists and activists who are immunising themselves from other viewpoints, or local experts who are not willing to consider any type of evidence that contradicts their personal experiences. Presumptuous scientific experts who see themselves beyond any doubt can also be cranks. The reason why cranks should be excluded is that their dismissive attitude towards open exchange undermines the Millian ideal that Feyerabend endorses: without the motivation to question one’s own ideas when considering other viewpoints, there can be no genuine deliberation and, hence, the proliferation of viewpoints is deprived of its merits. Although it provides no sharp demarcation criterion and is likely to be difficult to operationalise in practice, the “crank-test” can serve as useful design principle when thinking about who should be included in institutionalised policy advice.

All these objections point to intricate obstacles for implementing epistemic pluralism in science-based public health policy–and policy-making more broadly. While our answers will most likely not have removed all doubt, we tried to make it clear that a higher degree of epistemic pluralism is desirable in principle and that it is possible to realise more epistemic pluralism in science-based policy–*in effect transforming it into an inclusive form of evidence-based policy*–by actively designing institutions and deliberative platforms that reflect this very idea.

## Conclusion

In this paper, we have provided an analysis of the political response to the COVID-19 pandemic from the perspective of Paul Feyerabend’s pluralistic philosophy. We draw attention to Feyerabend’s systematic arguments in favour of epistemic pluralism within science and we show how these arguments may be extended to extra-scientific contexts and to the relationship between scientific and non-scientific perspectives on questions of societal relevance. Based on Feyerabend’s discussions of the epistemic benefits of pluralism within science as well as his arguments in favour of the inclusion of extra-scientific perspectives in decision-making, we were able to show that the lack of epistemic pluralism in the way that evidence was used to inform policy during the COVID-19 pandemic was indeed problematic, and we have discussed reasons for why a higher degree of pluralism in the public-health policies during the pandemic would have been desirable for epistemic as well as political reasons. We also discussed the implications of these considerations for science-based policy more generally by suggesting an extension of what may count as permissible evidence and arguing in favour of integrating science-external elements in decision-making. While the integration of multiple perspectives and various types of evidence might be theoretically desirable, we understand that it is associated with a plethora of practical challenges. However, these challenges are not unsurmountable, and we believe it is a worthwhile endeavour for scholars in various fields (philosophy, STS, political science etc.) to think about schemes that allow for more epistemic pluralism–and hence for better informed and more even-handed policy-making.

## Footnotes

1 The purpose of this paper is to refine our analysis in Lohse and Bschir (2020) and explore the identified problems in more detail.

2 This point was stressed by many supporters of evidence-based medicine, see e.g. this statement by the German Network for EBM from March 2020: https://www.ebm-netzwerk.de/en/media/media/statement-20200323-covid-19-english.pdf (accessed July 2022).

3 For example, policy recommendations by the *German National Academy of Sciences Leopoldina* did take into account the expertise of social and educational scientists. But it is striking that hardly any studies and empirical results from these disciplines were referenced in pertinent policy papers (Bogner & Menz, 2021).

4 Many scholars have rightly emphasised the political nature of scientific policy advice and stressed the role that value-laden judgements, politics and power play at the interface of science and policy (Bacevic, 2020; Jasanoff, 1998; Stevens, 2020). In this sense, policy never really “follows the science” in a strict sense–not even in a pandemic.

5 See Broadbent and Streicher (2022) and Smart et al., (2021) for a discussion of specific issues in non-OECD countries.

6 Notable exceptions are four monographs on Feyerabend by Couvalis (1989), Preston (1997), Farrell (2003) and Oberheim (2006), a series of collected works (Munévar 1991; Brown & Kidd 2016; Munévar et al., 2000; Bschir & Shaw 2021), as well as a growing number of research articles dealing with Feyerabend positions (for an overview on the recent Feyerabend reception see the Introduction in Bschir & Shaw 2021).

7 Examples are Hasok Chang’s *Is Water H*_*2*_*O?* (2012), which shares many overlaps with Feyerabend’s pluralistic view of science but mentions Feyerabend only very briefly; or John Dupré’s *The Disorder of Things* (1993), which mentions Feyerabend as a major source of inspiration, but rarely refers to Feyerabend works in the text. In Philip Kitcher’s *Science, Truth and Democracy* (2001), Feyerabend’s main works are mentioned but not properly engaged with (see Shaw, 2021a for a comparison of Kitcher and Feyerabend). In a collected volume on *Scientific Pluralism* for the Minnesota Studies in the Philosophy of Science (edited by Stephen Kellert, Helen Longino and Kenneth Waters 2006), Feyerabend is mentioned only once in a footnote. Kitcher’s *Science in a Democratic Society* (2011) and Helen Longino’s *Science as Social Knowledge* (1990) are rare exceptions of important contributions of the past decades that do engage with Feyerabend’s ideas. Longino’s social account of scientific objectivity is based on a fundamental insight that also rests at the core of Feyerabend’s pluralism, namely that the cultivation of a diversity of scientific perspectives has strong epistemic benefits and contributes to the robustness and credibility of scientific knowledge.

8 Feyerabend also mentions Mach, Boltzmann and Mill as sources of inspiration for pluralism (Feyerabend, 1970, p. 211). For more on the influence of Mill see below.

9 It remains controversial as to whether Feyerabend’s own claims about how epistemic pluralism can support political pluralism are conclusive. Our distinction between a theoretical and a political stage should mainly be understood in a temporal sense: the arguments for political pluralism appear later in Feyerabend’s writings than the arguments for theoretical pluralism.

10 For a detailed discussion of Kuhn’s theory see Hoyningen-Huene (1993).

11 It is worth noting that Feyerabend does not straightforwardly dismiss what he calls *the principle of tenacity* (i.e. “the advice to select from a number of theories the one that promises to lead to the most fruitful results, and to stick to this one theory even if the actual difficulties it encounters are considerable”, Feyerabend 1970, p. 203). Feyerabend believes that adopting the principle of tenacity also forces us to adopt a principle of proliferation. Ultimately, he argues against the temporal succession of stages of tenacity and stages of proliferation. For a discussion of the interplay between the principles of tenacity and proliferation see also Bschir (2015), Oberheim (2006, Ch. 7), Preston (1997, pp. 95–96).

12 It has been argued elsewhere that Feyerabend’s account draws heavily on elements in Popper’s philosophy (see Bschir, 2015; see also Collodel, 2016; Tambolo, 2015).

13 “We shall take it as falsified only if we discover a reproducible effect which refutes the theory. In other words, we only accept the falsification if a low-level empirical hypothesis which describes such an effect is proposed and corroborated. This kind of hypothesis may be called a falsifying hypothesis” (Popper, 1959/2002, p. 66).

14 Popper uses the example of Galileo’s law of free fall and Newtonian mechanics. He highlights the fact that the former is incompatible with the latter, because it implies that the acceleration of a body in free falls is constant, whereas the latter assumes that it depends on the inverse square of the distance between the earth and the falling body (see Popper, 1957/1979; see also Bschir, 2015).

15 Feyerabend never used the term though (see Bschir, 2015).

16 On Feyerabend’s notion of incommensurability see Oberheim and Hoyningen-Huene (1997) and Oberheim (2005)

17 For criticisms of Feyerabend’s arguments for pluralism see Laymon (1977) and Worrall (1978). For an extensive discussion of Feyerabend’s theoretical pluralism and its relation to his methodological pluralism see Preston (1997, Ch. 7).

18 It is noteworthy that similar ideas are brought up in current debates. Hasok Chang, for instance, has defended what he calls “active normative epistemic pluralism” also based on the insight that “each system develops under certain constraints, which may prevent the production of elements that would actually help its own progress” (Chang 2012, p. 281).

19 Feyerabend uses the term “traditions” to refer loosely to any sort of human ways of living with their own histories of norms and principles and ways of approaching life and reality. For a discussion of Feyerabend’s use of “traditions”, “world views” and “forms of live” see the final chapter in Farrell (2003).

20 For more on Feyerabend’s anti-scientism see Kidd (2016a, 2016b, 2021a).

21 See Roe (2021) for an account of how Feyerabendian citizen science might look like.

22 For more on Feyerabend’s account of scientific expertise see Brown (2021) and Shaw (2021b). Sorgner (2016, p. 118) rightly points out that Feyerabend’s demand of pluralistic expertise also draws on the ideal of political representation: “[…] all traditions and value-systems should be treated equally and have equal say in public debates. This is to ensure that all concerns of the people affected by a technical decision are respected in the process.”

23 Scientism often goes hand-in-hand or can be an expression of (neo-)colonial forms of knowledge production, a connection that is somewhat underdeveloped in Feyerabend’s thinking. See Ludwig et al. (2021) for the state of the art of the discussion.

24 In Feyerabend (1980) and Feyerabend (1981), he engages with Mill’s ideas in more detail. His high esteem of Mill is also clearly visible in a letter to Imre Lakatos from 21 February 1971 (see Feyerabend 1999, pp. 238–241). For a critical discussion of Feyerabend’s engagement with Mill see Staley (1999) and Jacobs (2003).

25 See also Kellert et al., (2006, p. vx): “A pluralist stance keeps in the forefront the fact that scientific inquiry typically represents some aspects of the world well at the cost of obscuring, or perhaps even distorting, other aspects”.

26 Thanks to Thomas Reydon for encouraging us to clarify these distinctions.

27 To give an example: In April 2021, the Swiss government decided against the recommendation of its scientific COVID-19 task force to ease restrictions that had been implemented during the second wave of the pandemic. At that point, models had predicted an increase of infections in Switzerland for May, June and July 2021 if restrictions would be removed. As it turned out, the increase in cases did not happen and it was admitted by the involved scientific experts that their recommendation was based on model-based projections that were false in hindsight.

28 Note that this does not imply that the deployed epidemiological models were useless for making policy decisions (see the critical discussion in Winsberg et al., 2020). Rather it means that we need to acknowledge limitations of these models and judge them in light of their intended purposes (e.g. developing “reasonable worst case scenarios” (Birch, 2021), or long-term projections)–and policy-making in light of the appropriate understanding of those limitations and purposes (see van Basshuysen & White, 2021).

29 Note that there is a trade-off between model proliferation and (methodological) economy that will need to be considered but cannot be solved in the abstract.

30 SIR-models represent the interaction of susceptible, infectious, and recovered/removed individuals or subpopulations to project transmission dynamics during a pandemic.

31 Many of these types of models are developed not only by epidemiologists but by interdisciplinary teams, which makes it hard to locate them within a specific discipline.

32 For more details on potential contributions of the social sciences to pandemic policy, see Lohse and Canali (2021).

33 Indeed, many social scientists tried to get involved during the COVID-19 crisis. But more often than not, their input was not taken up by policy-makers (see, e.g. Hadorn et al., 2022).

34 In fact, the significance of airborne transmission of SARS-CoV-2 has long been downplayed by key health authorities such as the WHO, see Greenhalgh et al. (2021) for a fascinating sociological explanation for this.

35 Reported numbers and percentages for Long Covid vary widely, ranging from 2% of COVID-19 patients to more than 50%, as its definition is controversial between different stakeholders and clinical investigation is still on-going, but it is safe to assume that hundreds of thousands of people are affected worldwide.

36 This result was communicated in Swiss media (https://www.20min.ch/story/haelfte-der-corona-toten-stirbt-im-altersheim-305332030482) and confirmed in personal communication with the authors on June 17^th^ 2021. However, the data is not publicly available, because it was created for internal purposes by the responsible organisation based on conversations with inhabitants and patients’ provisions.

37 See https://www.bag.admin.ch/dam/bag/de/dokumente/mt/k-und-i/aktuelle-ausbrueche-pandemien/2019-nCoV/bericht-todesfaelle-februar-2021.pdf.download.pdf/Bericht%20Todesf%C3%A4lle%20im%20Zusammenhang%20mit%20Covid-19%20in%20der%20Schweiz%20und%20im%20internationalen%20Vergleich.pdf (accessed July 2022).

38 Note, however, that much “fast science” research during the COVID-19 pandemic was suffering from lack of rigor too (see Harvard & Winsberg 2021).

39 It is worth noting at this point that Feyerabend’s silence on the practical implementation of pluralism is probably due to his belief that there is no general, abstract mechanism for realizing pluralism in all given contexts. Practical implementations of pluralism are highly context dependent. Many thanks to Jamie Shaw for highlighting this point.
